# Social participation of older people in urban and rural areas: Canadian Longitudinal Study on Aging

**DOI:** 10.1186/s12877-023-04127-2

**Published:** 2023-07-18

**Authors:** C. Allyson Jones, Gian S. Jhangri, Shelby S. Yamamoto, David B. Hogan, Heather Hanson, Mélanie Levasseur, Ernesto Morales, France Légaré

**Affiliations:** 1 Dept of Physical Therapy, Faculty of Rehabilitation Medicine, University of Alberta, 2-50 Corbett Hall, Edmonton, AB T6G 2G4 Canada; 2 School of Public Health, University of Alberta, Edmonton, AB Canada; 3 Division of Geriatric Medicine, Department of Medicine, Cumming School of Medicine, University of Calgary, Calgary, AB Canada; 4 Alberta Health Services Provincial Seniors Health and Continuing Care, Calgary, AB Canada; 5 School of Rehabilitation, Université de Sherbrooke, Sherbrooke, QC Canada; 6 Research Centre on Aging, Estrie Integrated University Health and Social Services, Centre—Sherbrooke Hospital University Centre, Sherbrooke, QC Canada; 7 Department of Rehabilitation, Université Laval, Quebec City, QC Canada; 8 Department of Family Medicine and Emergency Medicine, Centre De Recherche Sur Les soins et Les Services de Première Ligne de S’Université Laval (CERSSPL-UL), Université Laval, Quebec City, QC Canada

**Keywords:** Social participation, Older adults, Built environment, Community, CLSA

## Abstract

**Background and objectives:**

Although the positive influence of social activity on health is now well-established, a complex relationship exists among social participation, personal, social and the environment. Social participation of older adults was examined in rural and urban settings to identify features of the built-environment and perception of neighborhood specific to the locale.

**Research Design and methods:**

Using cross-sectional data from the Canadian Longitudinal Study on Aging (CLSA), we examined social participation and health of older people (65 + yrs) in relation to the built environment and sociocultural contexts for urban and rural areas. A social participation index was derived from responses on the frequency of participating in 8 social activities over the past 12 months. Personal, household and neighborhood indicators were examined to develop multivariable regression models for social participation in urban and rural cohorts.

**Results:**

No meaningful differences were seen with the frequency of social participation between rural and urban settings; however, the type of community-related activities differed in that a greater proportion of urban participants reported sports and educational/cultural events than rural participants. Service club activities were greater for rural than urban participants. Different neighborhood features were statistically significant factors in explaining social participation in rural than in urban locales, although transportation was a significant factor regardless of locale. Trustworthiness, belonging and safety were perceived factors of the neighborhood associated with higher social participation for rural participants.

**Discussion and implications:**

The relationship between home and health becomes stronger as one ages. Social and physical features of built environment specific to urban and rural settings need to be considered when implementing appropriate social activities for older people.

## Introduction

Social participation plays an instrumental role in quality of life as one ages [1]. While several conceptualizations of social participation are described in the literature, social participation can be defined as a person’s involvement in community activities that provide social interactions within the community or society [2, 3]. The importance of social participation cannot be underestimated as a key determinant of healthy aging. A meta-analysis of 148 articles reported a protective effect of greater social participation on mortality that was comparable to cessation of smoking [4]. Personal factors such as age, gender, and health status are related to social participation [5, 6], as are neighborhood and social environments [7, 8]. A complex relationship exists between various personal health factors, neighborhood environment and social participation [9, 10]. Several personal and interpersonal factors are associated with social participation, yet a person’s interactions with the environments they inhabit also determine social participation [11]. An emerging area of research is environmental gerontology [12] that recognizes environmental influences on health and well-being, which profoundly affect our available options and choices [11, 13].

As the population ages and, more importantly, as we gain a better understanding of optimal health, there is growing interest in the role of built environment to promote healthy aging. The notion of aging-in-place does not only includes the home but also aspects of the built environment [14]. Built environment is a broad term that typically includes buildings, spaces and products that are created or modified by people such as housing, transportation and neighborhood characteristics [15] which undoubtedly varies between urban and rural locales. It is a component of environmental health and is a key factor of public health [15]. The natural environment is tightly connected with the infrastructure of the built environment and impacts physical and mental health [16, 17]. Earlier studies have reported positive associations between physical activity, health and green space [18–20]. Urban green spaces modestly predicted the strength of social ties and sense of community in neighborhoods among US inner-city older people [21] and for older people in Vancouver, Canada [22]. Rural settings, however, comprise the natural environment.

In recent years, attention has been directed toward the built environment and its associations with physical and mental health [23–26], yet little attention has been directed toward the built environment and older people who wish to age-in-place and remain active in their communities. With a growing aging population, older people’s choice is to remain in their homes for as long possible. Greater proximity and accessibility of resources is associated with greater social participation [27–29]. In other words, the neighborhood appears to be closely related to social participation and overall quality of life among older people. Aging is a dynamic process in which the needs of the person change over time, yet the residence and neighborhood may not change in response to personal needs.

Rural and urban locales will also have different implications on social participation. Sparse evidence, however, exists as to the roles that urban and rural features of residences and neighborhoods contribute to facilitating or impeding social participation [7]. Within the Canadian context, approximately 23.2% of older adults reside in rural and remote communities [30] with the rural population aging faster than urban populations [31, 32]. Rural and remote communities tend to have limited social and physical infrastructure, and capacity (e.g. human, financial resources) yet encompass distinct social and environmental features [33] with strong social networks and participation [34]. Moreover, substantial heterogeneity exists among Canadian rural regions in terms socio-economic features, distance from urban centers, and accessibility of services [35] which need to be considered when planning for options for older adults who wish to age-in-place.

Using data from the Canadian Longitudinal Study on Aging (CLSA), we examined social participation and health of older adults who were 65 years of older, in relation to the urban and rural built environments. Individual, residence and perception of neighborhood features specific to rural and urban communities that were associated with social participation were identified.

## Method

### Data source and participants

The CLSA is a large national study that aims to further our understanding as to why some people age healthy and others do not [36]. At time of recruitment, a national stratified sample of 51,388 women and men between 45 and 85 years old were enrolled. Although the CLSA follows participants every three years until death or 2033, at the time of this current analysis, only baseline cross-sectional data collected between 2011 and 2015 were available. Details of the recruitment process and the study design have been described elsewhere [37, 38]. Briefly, the CLSA has two components: the *tracking component* that included 21,241 participants followed by computer-assisted telephone interviews only; and the *comprehensive component* that was comprised of 30,097 participants who were interviewed in person, and underwent in-depth physical assessments at local Data Collection Sites (DCS). To support research integrating the two components, a common set of information was collected on both samples. Individuals in the *tracking component* were randomly selected within age and sex strata from each of the ten Canadian provinces [37, 38]. Individuals selected for the *comprehensive component* were randomly selected from within 25–50 kms of one of 11 DCS in seven provinces.

**Fig. 1:**
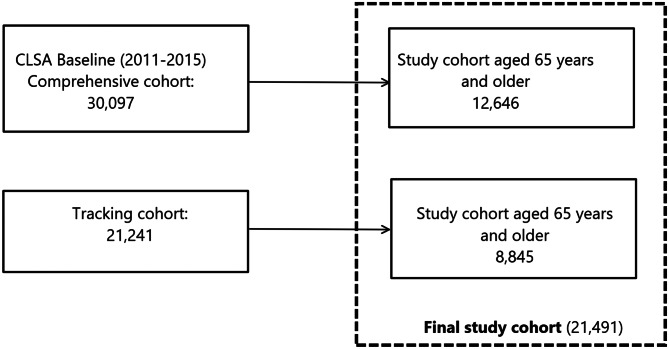
Flow chart of CLSA study cohort

Residents in the three territories and some remote regions, persons living in First Nations communities, full-time members of the Canadian Armed Forces, and individuals residing in long-term care institutions were excluded from the CLSA. Others who were unable to communicate in English or French, and those with cognitive impairment severe enough to preclude obtaining informed consent at the baseline interview were also excluded. Data used for this current analysis included participants from the *tracking* and *comprehensive* components who were 65 years and older at the baseline collection period from 2011 to 2015 (See Fig. 1 for flow chart of sample selection).

The framework for this analysis was guided by the International Classification of Functioning, Disability and Health (ICF) model [39] and the Aging in Place conceptual model [40]. Both frameworks acknowledge the complex process of the interrelationships between personal and environmental factors.

### Measures

#### Social participation

The primary outcome, social participation index was derived from responses on the frequency of participating in 8 social activities over the past 12 months. Specifically, these were activities with (1) family/friends, (2) sports/physical activities, (3) church/religious, (4) educational/cultural, (5) service club, (6) neighborhood/community/professional associations, (7) other recreational activities, and (8) volunteer/charity work. Responses for each of the social activities represented a score for the frequency of the social activities over the past 12 months (0 = *never*; 1 = *at least once per year*; 2 = *at least once per month*; 3 = *at least once per week*; 4 = *at least once per day*). These responses for each of the 8 social and community-related activities are summed to generate a social participation index with higher scores indicative of more frequent social participation. The overall social participation index scores range from 0 to 32 and has a high internal consistency (Cronback’s alpha 0.81 to 0.91) [41]. These social participation activities have undergone rigorous development and testing, and have been used in nationally representative surveys [42]. Two preference-based social participation questions were also asked regarding whether participants would prefer to participate in more activities, and if so, what prevented them from participating in more activities.

*Built environment*: The built environment was defined in terms of physical features of housing whereas, the social features of the environment consisted of the perception of the neighborhood [43]. Three specific environment questions were asked in the “Maintaining Contact” Questionnaire Wave 1 Version: (1) home and housing consisted of the type of housing, (2) length of residence, and (3) housing satisfaction. Two house-related questions dealt with physical problems encountered with the house (e.g., heating, condensation, repairs). Because the built environment is tightly connected with the infrastructure of the neighborhood including transportation, questions regarding the transportation use and frequency of public transit use in past 12 months were included.

The perception of the neighborhood consisted of a question that asked participants, “How do you feel about your local area, that is, everywhere within a 20-minute walk or about a kilometer from your home?” with respect to 9 situations that dealt with safety (e.g., walking in the dark; friendliness of people in the community), social cohesion (not feeling lonely, friendliness) and aesthetics of the neighborhood (e.g. cleanliness; vandalism/ graffiti).

Because urban and rural communities have different resources and facilities, we stratified residence locale specifically to examine social participation and built environments in rural and urban settings. Rural population consisted of the population residing outside of the urban centers [44, 45]. Although several definitions of rurality exist [46], we used the population metrics as defined by Statistics Canada consisting of urban setting including metropolitan (≥ 150,000 inhabitants), and urban (< 150,000 to ≥ 10,000 inhabitants) centers and the rural setting including those areas with less than 10,000 inhabitants [44].

*Social support*: Social features were estimated by the size and type of social network and functional social support which included the perceived availability of social support (Medical Outcomes Study (MOS) Social Support Survey) [47]. Social networks address the social connectedness of people’s social relationships [48]. The existing social network was defined in two ways: (1) *close social network size* which was based on number of family, friends and neighbors, and (2) *distant social network size* which was derived from the number of people at school, with community involvement, and/or at other activities.

Perceived availability of social support was assessed using MOS Social Support Survey which is a the 19-item, self-administered measure [47]. An overall score and 4 subscale scores (tangible- 4 items; affectionate support- 3 items; positive social interaction- 4 items; emotional support- 8 items) are generated with scores ranging from 0 to 100. Higher scores are indicative of greater social support [47, 49].

#### Health-related factors

Several factors were considered when examining overall health including 34 self-reported chronic medical conditions and functional capacity [7, 50]. The number and type of chronic conditions were examined in relation to social participation. The Older Americans Resources and Services (OARS) Multidimensional Assessment scale consists of 14 items measuring basic and instrumental activities of daily living (ADL, IADL) [51]. Responses were dichotomized as having no functional impairment or having impairment. Depressive symptomology was measured using 10-point cut point for the Center for Epidemiologic Studies Short Depression Scale (CESD-10) [52]. Socio-demographic information collected included demographics and lifestyle behaviors, physical/health-related measures, psychological measures, social heath and economic measures, and use of health services [37, 38].

### Statistical analysis

Descriptive analyses were performed for all variables to examine the distribution and outliers for the overall cohort, and then stratified for urban and rural locales. Because of the complex sampling design, sampling weights derived by CLSA were used to correct for possible differences in the sample from the reference population [53]. When estimating the mean value or proportion, inflation weights adjust the value so that it is representative of the provincial and national populations.

Based on these theoretical models, the independent variables examined individual, household and neighborhood indicators. Individual indicators included socio-demographic, health and lifestyle factors, whereas household indicators concerned such features as the house, number of people residing within home and ownership. The perceived neighborhood indicators included individual perceptions of the safety, belonging, trustworthiness and aesthetics of the community, transportation and duration residing within the neighborhood. This approach allowed explanation of intergroup variation by higher level variables including the individual-level, household-level and neighborhood-level covariables.

Owing to the approximately normal distribution of the dependent variable, social participation index, multiple linear regression analyses were performed to examine the associations between the independent variables and the dependent variable, social participation index separately for urban and rural cohorts. Variables found to be statistically significant at *p* < 0.2 at the univariate analysis were included in the first model as covariates [54]. Age and sex were considered important determinants of social participation index and were therefore included in all first and subsequent models, regardless of their level of statistical significance. In subsequent models, the variables with the highest *p*-values were eliminated sequentially, using stepwise backward elimination method. Confounding, set at a threshold of ≥ 15% change in regression coefficient, was investigated in each re-estimated model. Whenever a confounding relationship occurred between any two variables, both were retained in the model, and the variable with the next highest statistically non-significant *p*-value was considered for elimination. The final parsimonious models for urban and rural cohorts included statistically significant variables (*p* < 0.05), and all models were adjusted for age and sex. The variables not selected in initial selection of p > 0.2 were added to the final parsimonious model, one at a time, and checked for significance. This step is important for identifying variables that, by themselves, were not significantly related to the social participation index but make an important contribution in the presence of other variables in the model. None of these variables found to be statistically significant (p < 0.05) in the final parsimonious models [54]. After running the regression models, we also examined the standard error of each independent variable to decide whether the precision was adequate or not. None of the variables had large standard errors.

To account for sample misrepresentation related to unequal sampling probabilities and non-response, sampling weights [55] were used for all univariate and multivariable analyses as per CLSA guidelines. The variables that had greater than 5% missing values (income 8.3%, years spent in current community 8.8%, respondent felt about the 9 neighborhood questions 9.2–11.9%, and transportation 35.3%) were replaced with “not stated”. Other variables had less than 1% missing cases, except life satisfaction (1.8%). A listwise deletion was used for all independent variables with missing values less than 1%, and a total of 2.7% urban cases and 1.9% rural cases were excluded in multivariable analyses. Analyses were performed using STATA 17 statistical package [56]. The research analysis was conducted with the approval of the Health Research Ethics Review Board at the University of Alberta, Canada (Pro00075441).

## Results

Of the 21,491 CLSA participants aged 65 years and older in this analysis, 78.8% resided in urban areas and 21.2% from rural locations. Overall, the mean age of participants was 72.8 (95%CI 72.7, 72.9) years, with a larger proportion of urban participants (40.9%) 75 years or older as compared to rural participants (33.2%). Participants were predominantly female (53.5%). A greater proportion of rural residents were married or in common-law relationships (72.8%) compared to urban participants (64.8%) (Table 1). Differences between urban and rural locales were seen with socioeconomic indicators. A larger proportion of urban participants had university education and higher annual household income, whereas a higher proportion of rural residents owned their home. No differences between urban and rural residents were seen in health status with the majority reporting *good* or *very good* health (Table 1). While no functional impairments were reported in 84% in both urban and rural participants, 89% reported having two or more chronic conditions. The three most prevalent conditions reported in both cohorts were arthritis (39%), heart disease (27%) and diabetes (21%). A positive screen for depression was seen in 16.4% of the overall cohort. Lifestyle behaviors such as smoking and drinking alcohol were comparable between urban and rural participants.

**Table 1:** Sociodemographic, health, lifestyle and social characteristics of study cohort, overall and stratified by locale

	Overall	Urban	Rural	
**Characteristics**	(n = 21,491)	(n = 18,744)	(n = 2,747)	*p*-value
**Sociodemographic**				
Age groups, years				< 0.001
65–74	60.7	59.1	66.8	
75+	39.3	40.9	33.2	
Age, years, mean (95%CI)	72.9 (72.7, 73.0)	73.1 (72.9, 73.2)	72.1 (71.8, 72.4)	< 0.001
Retirement age, years, mean (95%CI)	61.1 (60.9, 61.3)	61.2 (61.0, 61.4)	60.7 (60.1, 61.2)	0.104
Sex				0.579
Male	46.5	46.4	47.2	
Female	53.5	53.6	52.8	
Marital status				< 0.001
Married/common-law relationship	66.5	64.8	72.9	
Single/divorced/separated	15.4	16.7	10.5	
Widowed	18.1	18.5	16.6	
Education				< 0.001
High school not completed	13.4	12.2	17.7	
Completed high school	13.3	12.7	15.4	
Some post-secondary	39.9	40.0	39.4	
University	33.5	35.1	27.5	
Annual household income				< 0.001
< $50,000	43.5	42.4	47.7	
$50,000- <$100,000	35.0	34.9	35.7	
> $100,000	13.2	14.1	9.7	
Not stated ^a^	8.3	8.6	6.9	
**Health**				
General health (self-rated)				0.563
Excellent	18.5	18.4	18.7	
Very good	38.8	38.4	40.4	
Good	30.0	30.3	28.5	
Fair	10.3	10.4	10.2	
Poor	2.5	2.5	2.2	
Functional impairment, OARS	15.5	15.9	13.8	0.050
Chronic condition				
Arthritis	39.5	39.1	40.9	0.232
Heart	26.6	26.8	25.7	0.386
Diabetes	20.7	20.7	20.7	0.993
Respiratory	15.6	15.7	14.9	0.428
Stroke	7.4	7.9	5.8	0.005
Cognitive	2.2	2.2	1.7	0.256
Parkinson	0.7	0.7	0.6	0.752
Chronic diseases index, mean (95%CI)	4.8 (4.8, 4.9)	4.9 (4.8, 5.0)	4.7 (4.6, 4.9)	0.149
Depression, CESD 10 + score, mean (95%CI)	5.3 (5.2, 5.4)	5.3 (5.2, 5.4)	5.2 (5.0, 5.5)	0.534
Positive screen for depression	16.4	16.4	16.5	0.923
Body mass index, BMI				0.865
Normal/underweight (< 25 kg/m^2^)	37.1	37.0	37.5	
Overweight (25-29.9 kg/m^2^)	39.7	39.7	39.8	
Obese (class I, II, III) (30 + kg/m^2^)	23.2	23.3	22.7	
**Lifestyle**				
Smoking status				0.768
Never	30.1	30.3	29.6	
Former	63.6	63.5	63.7	
Current	6.3	6.2	6.7	
Alcohol consumption				0.672
No	17.1	17.0	17.4	
Yes	82.9	83.0	82.6	
Life satisfaction				0.001
Yes	87.9	87.2	90.6	
No	10.3	10.9	8.0	
Not stated	1.8	1.9	1.3	
**Social Network**				
Number of living children				< 0.001
None	9.1	9.6	7.5	
1–3	66.5	67.1	64.2	
4+	24.4	23.4	28.3	
**Social Support**, mean (95%CI)				
MOS overall social support (SS) score,	82.4 (82.0, 82.7)	81.8 (81.4, 82.2)	84.4 (83.6, 85.3)	< 0.001
Tangible SS score	82.6 (82.1, 83.0)	81.8 (81.3, 82.3)	85.5 (84.5, 86.5)	< 0.001
Affection SS score	86.3 (85.9, 86.8)	85.9 (85.5, 86.4)	87.8 (86.8, 88.8)	< 0.001
Positive SS interaction score	82.8 (82.4, 83.3)	82.4 (82.0, 82.9)	84.3 (83.3, 85.3)	0.001
Emotional & informational SS score	80.4 (79.9, 80.8)	79.8 (79.4, 80.3)	82.3 (81.3, 83.3)	< 0.001

Note: Proportions (%) reported unless mean (95%CI) stated. CLSA sampling weights were applied to all analyses to adjust for sampling probabilities

a More than 5% cases had missing values and were replaced with “not stated.” For other variables missing values were < 2%

Abbreviations: OARS, Older American Resources and Services Multidimensional Assessment scale; CES-D, Center for Epidemiologic Studies Short Depression Scale; BMI, Body Mass Index; MOS, Medical Outcomes Study Social Support Survey

When considering social networks, more rural residents reported a network of relatives, neighbors and close friends (45.7%) than urban participants (40.1%). Distant social networks, which were defined as people known through community and association involvement, were reported by more rural participants (63.5%) compared to urban (59.3%) residents. A greater proportion of rural participants were living with others (78.1%) than urban (69.6%) participants. Rural residents reported a greater number of neighbors and living children than urban participants (p < 0.001). (Table 1)

Rural participants had greater perceived social support (higher MOS mean overall scores 84.4; 95%CI: 83.6, 85.3) compared to urban participants (81.8; 95%CI: 81.4, 82.2) (p < 0.001). (Table 1) Among the four MOS subscales, the highest mean score was seen for the affectionate support subscale (mean score = 86.3%, 95%CI: 85.9 to 86.8). In all four MOS subscales, the mean scores were slightly higher in rural residents, with the largest urban-rural difference found with the tangible support score (urban 81.8; 95%CI: 81.3, 82.3; rural 85.5; 95%CI: 84.5, 86.5; p < 0.001).

### Social participation

**Fig. 2:**
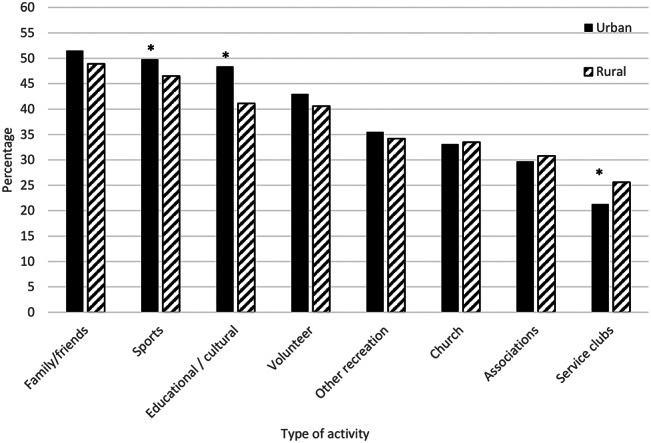
Frequent participation in social activities by residence region. Frequent participation refers to ‘at least once/week’ participation in more common activities (outdoor activities with friends and family, sport or physical activities, church or religious activities such as services, committees or choirs, and other recreational activities involving other people such as hobbies, gardening, poker, bridge, cards, and other games), and ‘at least once/ month’ participation in less common activities (such as attending courses, concerts, plays, or visiting museums), neighborhood/ community/ professional association activities, and service club or fraternal organizational activities, and volunteer or charity-related activities [5] ^*^ *Significant difference (p < 0.05)*

The mean social participation index of this cohort was 11.3 (95% CI: 11.1 to 11.4) with no large meaningful difference between urban and rural participants (Table 2). As seen in the frequency distribution for the 8 types of social participation (Fig. 2), the proportion of urban participants was greater for educational /cultural events and sporting activities than rural participants. Urban participants, however, participated in less service club/organization work than the rural participants (Fig. 2). Interestingly, 33.8% of urban participants expressed a desire to participate in more social activities as compared to rural participants (29%). The most common response by those who expressed no interest in being more social active was “being too busy”.

**Table 2:** Social participation of overall study cohort and stratified by locale

	Overall		Urban	Rural	
**Characteristics**	(n = 21,491)		(n = 18,744)	(n = 2,747)	*p*-value
Social participation index, mean (95%CI)	11.3 (11.1, 11.4)		11.3 (11.2, 11.4)	11.0 (10.7, 11.3)	0.034
Desire to participate in more activities,	32.8		33.8	29.0	< 0.001
Reason for not participating in more activities (% of ‘yes’ responses only)					
Being too busy	30.9		30.5	32.6	0.408
Health condition/ limitation	23.5		24.2	20.4	0.102
Personal/ family responsibility	15.1		15.0	15.5	0.812
Going alone	13.7		14.0	12.6	0.481
Suitability of activity timing	8.7		8.5	9.8	0.424
Other reasons	11.7		11.7	11.6	0.867

Note: Proportions (%) reported unless mean (95%CI) mentioned. CLSA sampling weights were applied to all analyses to adjust for sampling probabilities

### Built environment

Differences existed between urban and rural locales with respect to the physical aspects of housing. In particular, the majority of rural participants (91.2%) owned a house as compared to those who resided in urban centers (82.4%) with the most common type of dwelling being a house (Table 3). Regardless of locale, participants had been in their current residence for more than 22 years and were satisfied with the residence (97.3%). Most participants (82.3%) reported no problems with their houses (Table 3). To this end, almost all participants in this cohort were satisfied with their current housing with less than a one-fifth reporting problems with it (Table 3).

**Table 3:** Housing, neighborhood features, and transportation for overall cohort and stratified by locale

			Overall	Urban	Rural	
**Characteristics**			(n = 21,491)	(n = 18,744)	(n = 2,747)	*p-*value
**Housing**						
House ownership						< 0.001
Own			84.2	82.4	91.2	
Rent			15.8	17.6	9.8	
Dwelling type						< 0.001
House			77.5	73.1	93.8	
Not a house			22.5	26.9	6.2	
Number of people living in the household						< 0.001
None			27.8	29.4	21.9	
1–2			69.2	67.6	75.4	
≥ 3			3.0	3.0	2.7	
Years spent in current home, mean (95%CI)			22.9 (22.5, 23.2)	22.5 (22.1, 22.9)	24.1 (23.2, 25.1)	0.002
Reasons for moving to current location						0.012
Personal/family related			43.6	43.1	45.6	
Housing related			35.0	35.5	32.6	
Availability of services			6.9	7.2	5.5	
Other			14.7	14.2	16.3	
Satisfied with current housing			97.3	97.4	97.2	0.682
Problems with current home						0.400
Yes problem			17.7	17.5	18.5	
No problem			82.3	82.5	81.5	
**Neighborhood**						
Yrs. spent in current community, mean (95%CI)			35.3 (34.8, 35.8)	35.8 (35.3, 36.4)	33.5 (32,2, 34.7)	< 0.001
Yrs. spent in current community group						< 0.001
≤24			31.8	30.4	37.3	
25–45			31.2	32.2	27.6	
> 45			28.1	28.7	26.1	
Not stated ^a^			8.8	8.7	9.0	
Respondents ^b^ agreed to statements:						
most people in local area are friendly			98.3	98.2	98.7	0.164
local area is kept very clean			97.4	97.2	98.0	0.090
people in local area will not take advantage of them			96.8	96.6	97.3	0.252
most people in local area can be trusted			96.8	96.5	97.9	0.009
lots of people in local area who would help if in trouble			96.4	96.2	97.0	0.172
vandalism or graffiti are not a big problem in local area			95.2	94.5	97.9	< 0.001
a part of local area			94.7	94.4	95.6	0.097
not feeling lonely living in local area			91.6	91.7	91.1	0.444
not afraid to walk alone after dark in local area			88.8	87.7	88.0	0.772
**Transportation**						
**Most common form of transportation in the past year**						
Self-driven ^c^			58.4	58.2	59.2	0.002
Other modes			6.3	5.8	8.2	
Not stated ^a^			35.3	36.0	32.5	

Note: Proportions (%) reported unless mean (95%CI) stated. CLSA sampling weights were applied to all analyses to adjust for sampling probabilities

a More than 5% cases had missing values and were replaced with “not stated.” For other variables missing values were < 2%

b Missing values were 9.2–11.9% and were replaced with “not stated”, reported % are based on the non-missing cases

c Self-driven includes wheelchair/ motorized scooter/ cycling/ walking

The social dimensions of the neighborhoods which consisted of perceptions in terms of safety, social cohesion and aesthetics had an overwhelming sense of positive responses (Table 3). Participants, regardless of locale, had been residing in their neighborhoods for greater than 30 years (mean 35.3 years, 95%CI 34.8, 35.8). The most common form of transportation was self-driving (58.4%); however, rural participants (8.2%) reported taking taxis or being a passenger more often than urban participants (5.8%).

### Relationship of built environment with social participation

The perceived neighborhood features differed between urban and rural participants within the adjusted multivariable modelling (Table 4). Different neighborhood features were statistically significant factors in explaining social participation in rural than in urban locales, although transportation was a significant factor regardless of locale. A sensitivity analysis using only non-missing responses for transportation (n = 13,905) still identified transportation and its interaction with house ownership as significant factors for explaining social participation both in urban and rural locales.

**Table 4:** Multivariable analysis for urban and rural cohorts examining the association between the outcome ‘social participation index’ with personal, social network, house and neighborhoods characteristics

	Urban (n = 18,237)		Rural (n = 2,717)	
**Characteristics**	Coeff (95% CI)	*p*-value	Coeff (95% CI)	*p*-value
**Socio-demographic**				
Age groups (referent: 65–74 years)				
75 + years	-0.004 (-0.15, 0.14)	0.958	-0.21 (-0.60, 0.19)	0.309
Sex (referent: female)				
Male	-1.17 (-1.31, -1.02)	< 0.001	-1.45 (-1.84, -1.07)	< 0.001
Marital status (referent: married/common-law)				
Single/divorced/separated	-0.42 (-0.72, -0.13)	0.005	-1.12 (-1.74, -0.50)	< 0.001
Widowed	0.15 (-0.13, 0.44)	0.297	-0.25 (-0.78, 0.28)	0.366
Education (referent: completed high school)				
High school not completed	-0.66 (-0.94, -0.38)	< 0.001	-0.95 (-1.59, -0.32)	0.003
Some post-secondary	0.61 (0.40, 0.83)	< 0.001	0.20 (-0.34, 0.75)	0.463
University	1.58 (1.35, 1.81)	< 0.001	1.28 (0.70, 1.87)	< 0.001
Annual household income (referent: < $50,000)				
$50,000 - <$100,000	0.60 (0.42, 0.76)	< 0.001	-	
$100,000+	0.51 (0.28, 0.73)	< 0.001	-	
**Health**				
No functional impairment (referent: OARS: no)	-0.64 (-0.84, -0.46)	< 0.001	-0.84 (-1.38, 0.31)	0.003
Chronic condition				
Heart	-0.17 (-0.33, -0.01)	0.032	-	
Diabetes	-0.56 (-0.73, -0.39)	< 0.001	-	
Depression (referent: CESD < 10)	-0.69 (-0.89, -0.49)	< 0.001	-	
BMI (referent: normal/underweight, < 25 kg/m^2^)				
Overweight (25-29.9 kg/m^2^)	0.51 (0.35, 0.67)	< 0.001	-	
Obese (30 + kg/m^2^)	0.24 (0.06, 0.42)	0.011	-	
**Lifestyle**				
Smoking status (referent: never)				
Former	-0.50 (-0.65, -0.35)	< 0.001	-0.21 (-0.61, 0.20)	0.302
Current	-2.59 (-2.89, -2.28)	< 0.001	-2.19 (-3.01, -1.38)	< 0.001
Drinking status (referent: no)	0.49 (0.30, 0.67)	< 0.001	-	
No Life satisfaction (referent: yes)	-1.38 (-1.61, -1.15)	< 0.001	-1.37 (-2.02, -0.72)	< 0.001
**Social Network**				
Number of living children (referent: none)				
1–3	0.33 (0.09, 0.58)	0.007	-	
4+	0.63 (0.36, 0.90)	< 0.001	-	
**House**				
House ownership (referent: own)				
Rent	-0.34 (-0.62, -0.07)	0.015	0.07 (-0.90, 1.04)	0.887
Dwelling type other than a house (referent: house)	0.38 (0.19, 0.57)	< 0.001	-	
**Neighborhood**				
Number of people living in the household				
1–2	-0.15 (-0.43, 0.12)	0.266	-	
≥ 3	-0.66 (-1.11, -0.21)	0.004	-	
Respondent disagreed (referent: agreed)				
lots of people in local area who would help if in trouble	-0.74 (-1.12, -0.36)	< 0.001	-	
not feeling lonely living in local area	-0.75 (-1.03, -0.47)	< 0.001	-	
a part of local area	-1.42 (-1.75, -1.09)	< 0.001	-1.11 (-1.97, -0.25)	0.012
most people in local area can be trusted	-		-1.95 (-3.30, -0.59)	0.005
not afraid to walk alone after dark in local area	-		-0.80 (-1.39, -0.22)	0.007
**Transportation**				
Transportation in the past year other than self-driven	-0.81 (-1.12, -0.50)	< 0.001	-1.05 (-1.79, -0.32)	0.005
**Interaction**				
Transportation * House Ownership	1.02 (0.24, 1.80)	0.010	2.78 (0.36, 5.21)	0.024

Note: CLSA sampling weights were applied to all analyses to adjust for sampling probabilities

Dashes (-): Variable and/or its categories were non-significant (p > 0.05) in the multivariable model

Abbreviations: OARS, Older American Resources and Services Multidimensional Assessment scale; CES-D, Center for Epidemiologic Studies Short Depression Scale; BMI, Body Mass Index

For urban participants, social belonging were features associated with higher frequency of social participation. Trustworthiness, sense of belonging and safety were perceived neighborhood factors associated with higher social participation for rural participants. Socio-economic indicators such as level of education and home ownership were associated with higher frequency of social participation regardless of urban or rural locales. Functional impairment, being a smoker, and perceived less satisfaction with life were associated with lower social participation. Urban residents with children had less social participation outside of the home than participants without children whereas, this was not a significant factor for rural participants.

## Discussion

Social participation is not only an individual choice but one that is influenced by the built environment. Our findings based on a national study indicated that urban and rural specific features, housing and perception of the neighborhood play key roles in social participation of older people in Canada. Activities with family and friends, and volunteering were common types of social activities regardless of locale; however, rural residents were more active with service clubs than urban participants. Urban residents reported sporting, educational and cultural activities more often than rural people. Social dimensions of a neighborhood such as a sense of belonging in the community was associated with higher frequency of social participation in older people; however, perception of civic trust and safety were associated with social participation only in the rural cohort. Interestingly, perception of neighborhood safety was not a statistically significant factor for urban areas.

The relationship between home and health becomes stronger as one ages. Older people typically reside in older homes in older neighborhoods which frequently have environmental barriers for persons with limited mobility [25, 57, 58]. Accessibility is defined within this context as the inter-relationship between the demands of the physical environment and the person’s functional capacity[39, 59]. Similar to others [60], functional impairment was associated with less social participation regardless of locale. If duration in residence and community is reflective of accessibility within ones’ community, over half of our cohort resided in the community for 25 years or longer with over two decades spent in their current homes and were satisfied with their current housing regardless of locale. The majority of participants also reported *good* to *excellent* health with minimal functional impairments[38], and could drive a vehicle.

Regardless of locale, our findings found that transportation was an independent factor that explained social participation. Others have also identified transportation as an integral component of social activity for older people [43, 61]. This is a key factor to social participation, in particular for rural communities that do not have infrastructure for public transportation. For rural and remote communities, lower population density, lengthy travel, isolation and limited public transportation are features distinct to these locales [62] that impact social activity.

Maintaining independence and safety at home becomes more significant with age as functional independence declines with physiological aging. A key component to home and health is based on mutual participation, preferences, perceptions and social interactions. The home environment is not only the physical structure but, as Mahler and colleagues explain, it supports people as they age, provides close vicinity to family and creates a sense of neighborhood/community life [63]. Restriction in social participation has been attributed to several individual factors including loss of family and friends, lack of supportive community, awareness of social opportunities [64], and loss of mobility [65]. Social participation plays a protective role in the mental health of older adults. Isolation or limited social interaction predicts depressive symptoms [66]. Congruent with others, depression was a factor for both urban and rural cohorts that explained lower social participation in our multivariable models. In a younger Canadian cohort of participants 45 to 64 years of age, Griffin and colleagues also reported that depression alone or in combination with other chronic conditions was related to restricted social activities [50]. A similar relationship was also highlighted in a systematic review in that higher social activity reduced depressive symptomology over time [66].

Within this national study, we provide further evidence as to the frequency and type of social participation, and the associated built environmental features that are specific to urban and rural locales for older people in Canada. We used multivariable modelling which allowed us to evaluate the relationship among personal, social and built environment variables to develop a parsimonious models. Findings from this study should be viewed in light of a few methodological limitations. The relationship among housing/community and perception of neighborhood, social participation and health is a complex paradigm. Because this study was cross-sectional, causal inference cannot be drawn and only explanatory factors of social participation identified. Moreover, some of the associations may be bi-directional in that people with limited social participation may restrict their activities and healthy behavior. We were also constrained by the survey items for social participation and perceptions of the neighborhood. As definitions evolve, for instance as with social participation [3], consideration of the wording for questions need to be to reflective of the conceptual changes seen in the literature. We measured social participation using a validated index which quantified the frequency of predefined social activities [41] and did not capture other conceptual definitions of social participation such as the *where*, *when* and *why* as proposed by Levasseur and colleagues [3]. The frequency of social participating can document informal and voluntary social engagement but exclude common forms of social connectedness (phone calls, home visits, etc.) and does not support conclusions about the person’s performance when engaging in these activities. Providing other dimensions of social participation are warranted to provide fuller understanding of the association of social participation in urban and rural settings. As the longitudinal CLSA data collects data over time, this may also warrant further investigation of factors predictive of social participation.

Similar to the measurement of social participation, a consideration with these analyses concerned the constructs used to evaluate the built environment. For instance, a complex interplay between several dimensions of neighborhood safety and health outcomes of older people has been recognized yet more rigorous self-report and objectives measures of the neighborhood are called for by others [67, 68]. Within the CLSA, few questions dealt with the built environment which may limit the association of the built environment in explaining social participation. Several physical environmental factors exist when evaluating a neighborhood, such as functionality, safety aesthetics and destination, yet not all of these constructs of a built environment were captured in the CLSA. Because of the interest in ecologic determinants of health, particularly in vulnerable populations such as older people, others have called for more rigorous studies and measures of built environments in the literature [67, 69, 70].

Aging-in-place is an underlying principle which is motivated both by quality of life and economic intentions of which social participation is one feature. Rural and urban communities have distinct characteristics which need to be recognized when considering aging-in-place. Housing and perception of the neighborhood are significant factors in terms of social participation for older people, who often spend more time in their communities. Evaluating social and physical features of built environment are key aspects that need to be considered when determining whether older adults will remain socially active in their communities. These features, however, are different for urban and rural locales which have implications on social and environmental planning for older adults residing in these distinct regions. Further investigation of longitudinal data is warranted to identify determinants of other built environment features on social participation over time.

## Data Availability

Data are available from the Canadian Longitudinal Study on Aging (www.clsa-elcv.ca) for researchers who meet the criteria for access to de-identified CLSA data.

## Abbreviations

ADL: Activities of daily living
CLSA: Canadian Longitudinal Study on Aging
CESD: Center for Epidemiologic Studies Short Depression Scale
DCS: Data Collection Sites
IADL: Instrumental activities of daily living
ICF: International Classification of Functioning, Disability and Health
MOS: Medical Outcomes Study
OARS: Older Americans Resources and Services
